# Patient-level costing for the Thai Diagnosis Related Group in Thailand: a micro-costing approach

**DOI:** 10.1186/1472-6963-11-S1-A2

**Published:** 2011-10-19

**Authors:** O Khiaocharoen, S Pannarunothai, C Zungsontiporn, A Riewpaiboon

**Affiliations:** 1 Phitsanulok Provincial Health Office, Muang, Phitsanulok, 65000, Thailand; 2 Centre for Health Equity Monitoring (CHEM), Faculty of Medicine, Naresuan University, Muang, Phitsanulok, 6500, Thailand; 3 Central Office for Healthcare Information, Health Systems Research Institute (HSRI), Nonthaburi, 11000, Thailand; 4 Faculty of Pharmacy, Mahidol University, Bangkok, 10400, Thailand

## 

Cost estimation is important in assessing a health system’s performance. However, most of the costing system that presently exists presumes that all patients consume exactly the same amount of resources, and little attention is paid to costs at the patient level. Thailand has used Thai DRG for the prospective payment of inpatient care with a closed end, but there is a growing need to have patient-level cost data to calculate relative weight.

This report presents a brief summary of the technical details involved in patient-level costing for Thai DRG version 5. Cost methodology focused on a provider perspective, and cost data were collected from nine hospitals in the North, Central and Northeast of Thailand. These comprised two medical school hospitals, three community hospitals, two provincial hospitals, and two regional hospitals.

The primary data collected included the proportion of working time to apportioned labour cost, patient demographic characteristics, and medical data from 349,275 inpatients. Secondary data included hospital expenditures and the total number of medical services provided by each hospital unit in the fiscal year 2009. Cost analyses consisted of four major processes.

First, hospital cost was analysed using a standard top-down approach. Cost centre identification, direct, indirect, and total cost determination for 14 chargeable service units were examined. Second, the cost-to-charge ratio (RCC) was calculated by dividing the total cost by the total charge for each of 14 service groups. Third, a micro-costing method was employed for patient-level costing. To determine cost, the charge of each service group was converted to a cost by multiplying the charge by the corresponding RCC. This was then summed up to derive the total cost for each patient. Finally, all patient data were grouped into Thai DRG version 5, and then the average cost per admission, average cost per DRG, and RW were calculated.

## Recommendation

Micro-costing with a cost-to-charge ratio can be used for cost estimation and calibration of the relative weight of a DRG to establish a hospital payment policy.

